# Micro-Transcriptome Analysis Reveals Immune-Related MicroRNA Regulatory Networks of *Paralichthys olivaceus* Induced by *Vibrio anguillarum* Infection

**DOI:** 10.3390/ijms21124252

**Published:** 2020-06-15

**Authors:** Xianhui Ning, Li Sun

**Affiliations:** 1 CAS Key Laboratory of Experimental Marine Biology, Center for Ocean Mega-Science, Chinese Academy of Sciences, Institute of Oceanology, Qingdao 266071, China; xhningouc@163.com; 2 Laboratory for Marine Biology and Biotechnology, Qingdao National Laboratory for Marine Science and Technology, Qingdao 266237, China

**Keywords:** microRNA, *Paralichthys olivaceus*, *Vibrio anguillarum*, immune pathway, miRNA–mRNA interaction network

## Abstract

MicroRNAs (miRNAs) are non-coding regulatory RNAs that play a vital part in the host immune response to pathogen infection. Japanese flounder (*Paralichthys olivaceus*) is an important aquaculture fish species that has suffered from bacterial diseases, including that caused by *Vibrio anguillarum* infection. In a previous study, we examined the messenger RNA (mRNA) expression profiles of flounder during *V. anguillarum* infection and identified 26 hub genes in the flounder immune response. In this study, we performed the micro-transcriptome analysis of flounder spleen in response to *V. anguillarum* infection at 3 different time points. Approximately 277 million reads were obtained, from which 1218 miRNAs were identified, including 740 known miRNAs and 478 novel miRNAs. Among the miRNAs, 206 were differentially expressed miRNAs (DEmiRs), and 104 of the 206 DEmiRs are novel miRNAs identified for the first time. Most of the DEmiRs were strongly time-dependent. A total of 1355 putative target genes of the DEmiRs (named DETGs) were identified based on integrated analysis of miRNA-mRNA expressions. The DETGs were enriched in multiple functional categories associated with immunity. Thirteen key DEmiRs and 66 immune DETGs formed an intricate regulatory network constituting 106 pairs of miRNAs and DETGs that span five immune pathways. Furthermore, seven of the previously identified hub genes were found to be targeted by 73 DEmiRs, and together they formed interlinking regulatory networks. These results indicate that *V. anguillarum* infection induces complicated miRNA response with extensive influences on immune gene expression in Japanese flounder.

## 1. Introduction

MicroRNAs (miRNAs) of ~22 nucleotides (nt) represent the most well characterized small non-coding RNAs that regulate many fundamental biological processes such as growth, reproduction, and immunity [1,2]. The typical mechanism of miRNA regulation is by targeting protein-coding genes at the post-transcriptional level, resulting in mRNA degradation or translational suppression [3]. In recent years, the identified targets of miRNAs have been extended to non-coding RNAs such as long non-coding RNAs and circular RNAs, which indicates a crucial role of miRNAs as bridges linking different types of RNA molecules into complex interacting networks [4,5]. In addition, miRNAs can also serve as novel diagnostic biomarkers and therapeutic targets for pathogenic diseases [6,7].

Small RNA-sequencing (sRNA-seq) is an efficient technology to systemically study the expression profiles of miRNAs under various conditions. Benefiting from this technique, many fish miRNAs involved in the immune response associated with pathogen infection have been identified. These miRNAs include the common carp (*Cyprinus carpio*) miRNAs induced by *Flavobacterium columnare*, Nile tilapia (*Oreochromis niloticus*) miRNAs induced by *Streptococcus agalactiae*, and half-smooth tongue sole (*Cynoglossus semilaevis*) and miiuy croaker (*Miichthys miiuy*) miRNAs induced by *Vibrio anguillarum* [8,9,10,11]. In Japanese flounder (*Paralichthys olivaceus*), sRNA-seq has been used to explore miRNAs participating in the infections of megalocytivirus, *Edwardsiella tarda*, and *Streptococcus iniae* [12,13,14]. These studies demonstrated that pathogens could significantly affect the expressions of large amounts of host miRNAs [15,16,17].

Japanese flounder is an important aquaculture fish with great economic values. It is susceptible to *V. anguillarum*, the etiological agent of vibriosis. Vibriosis is one of the most common aquatic diseases and has caused huge economic losses worldwide [18,19]. To date, the immune mechanism of flounder against *V. anguillarum* is largely unclear. Previous studies on anti-*V. anguillarum* immunity of flounder focused mainly on protein-coding genes [20,21,22]. Recently, we have investigated the messenger RNA (mRNA) expression profiles of flounder induced by *V. anguillarum* [23]. However, no study on miRNA-based micro-transcriptome of flounder associated with *V. anguillarum* has been documented.

In this study, we performed the micro-transcriptome analysis of flounder in response to *V. anguillarum* infection at three different time points (6 h, 12 h and 24 h). The miRNA that exhibited differential expression (named DEmiRs) during *V. anguillarum* were characterized. The target genes of the DEmiRs (named DETGs) were identified based on the integrated analysis of miRNA-mRNA expressions. The DETGs were enriched functionally, and immune-related DEmiR-DETG networks were constructed and analyzed. Our study provides the first systemic micro-transcriptome data of flounder induced by *V. anguillarum*, and will be useful for future investigations on the immune mechanisms of miRNAs in flounder.

## 2. Results

### 2.1. Data Processing and miRNA Identification

**Figure 1:**
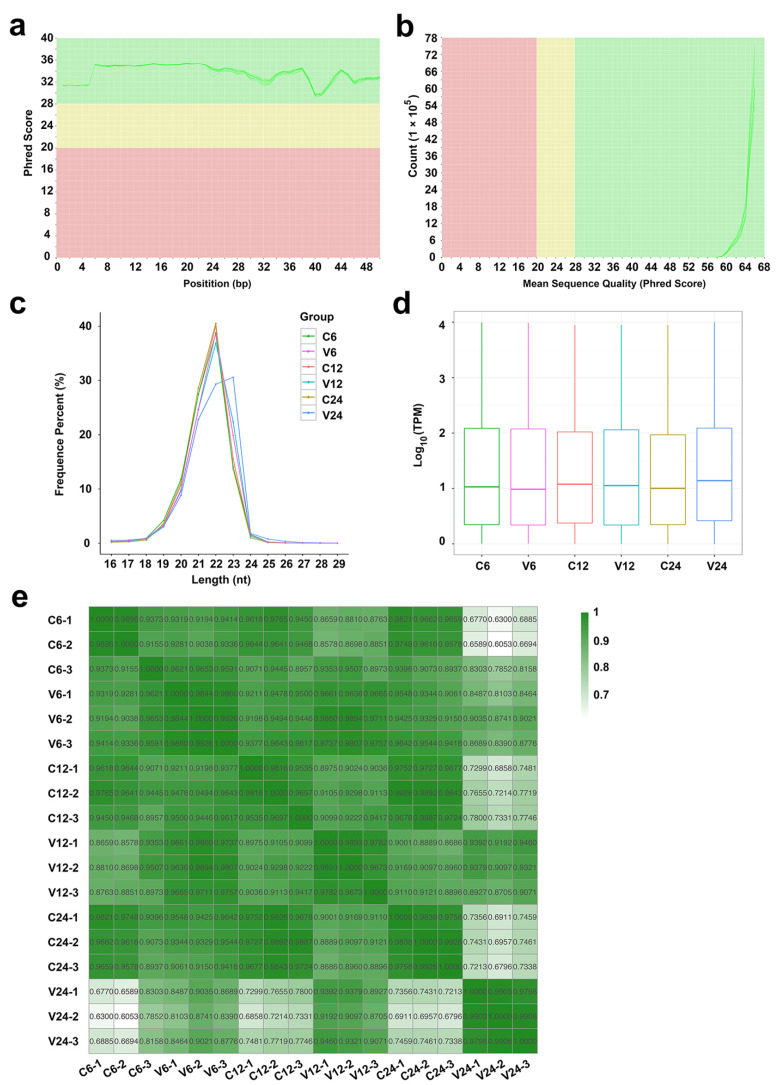
Sequencing data quality control and miRNA feature identification. (**a**) The mean quality scores of the small RNA-seq (sRNA-seq) data. bp, base pair. (**b**) The per sequence quality scores of the sRNA-seq data. (**c**) The length distribution of the miRNAs in different groups. For convenience, “C6”, “C12”, and “C24” indicate the control groups at 6, 12, and 24 h post-infection (hpi), respectively; “V6”, “V12”, and “V24” indicate *Vibrio anguillarum*-infected groups at 6, 12, and 24 hpi, respectively. nt, nucleotide. (**d**) Boxplot showing the expression patterns of the miRNAs in different groups at different time points. TPM, transcripts per million. (**e**) The correlation matrix of the miRNA expressions among the samples of different groups at different time points. For each group at each time point, three individual fish (indicated by the postfix number of 1, 2, or 3) were used. The correlation coefficients among the samples are indicated by numbers.

A total of 276,860,895 raw reads were obtained from the 18 sequencing libraries, 93.51% of which passed the filtering processes and were identified as clean tags (Table 1). FastQC analysis showed that these tags had high qualities with the mean quality scores higher than 28 and the per sequence quality scores higher than 58 (Figure 1a,b), indicating that they were suitable for subsequent analysis. Among the clean tags, 94.19% were aligned to known miRNAs, 0.27% were predicted to be novel miRNAs, 2.71% were identified as other sRNAs, containing ribosome RNAs (rRNA), transfer RNA (tRNA), small nuclear RNA (snRNA), small nucleolar RNA (snoRNA), and small cytoplasmic RNA (scRNA), and the remaining 2.83% were tags including unannotated sequences and repeated sequences. In total, 1218 miRNAs were identified, of which, 740 are known miRNAs and 478 are novel miRNAs discovered for the first time. The length distribution of the majority of the miRNAs is in the range of 20–23 nt, mostly 22 nt, except for the miRNAs of the V24 group, which are predominantly 23 nt (Figure 1c). The expression profiles of the miRNAs in the control and *V. anguillarum*-infected fish at each time point are shown in a boxplot (Figure 1d). At each time point, the miRNA expressions in the biological triplicates displayed correlation coefficients >0.9, indicating a high repeatability of the samples in each group (Figure 1e).

### 2.2. Identification and Validation of DEmiRs Induced by V. anguillarum

**Figure 2:**
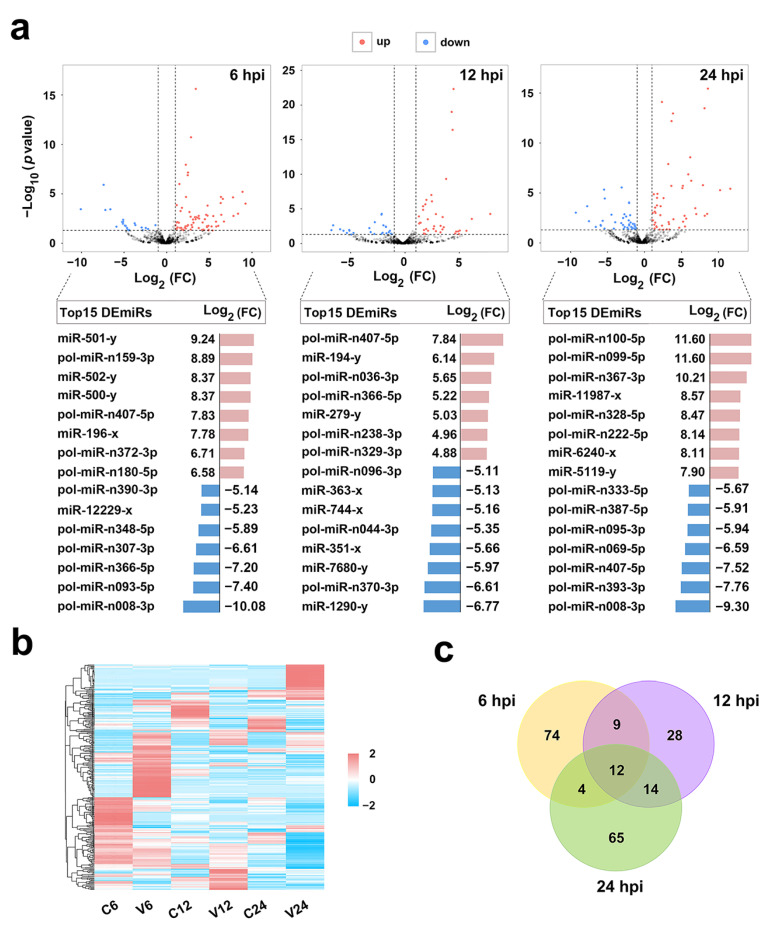
Expression patterns of the differentially expressed miRNAs (DEmiRs). (**a**) Volcano plots of DEmiRs at 6, 12, and 24 h post-infection (hpi). “up” and “down” indicate up- and down-regulated DEmiRs, respectively. FC, fold change. The top 15 up- and down-regulated DEmiRs and their FC in expression at each time point are shown below the corresponding volcano plot. (**b**) Heat-map showing the expression profiles of the DEmiRs in different groups at different time points. For convenience, “C6”, “C12”, and “C24” indicate the control groups at 6, 12, and 24 hpi, respectively; “V6”, “V12”, and “V24” indicate the *Vibrio anguillarum*-infected groups at 6, 12, and 24 hpi, respectively. (**c**) Venn diagram showing overlapping DEmiRs at different time points.

**Figure 3:**
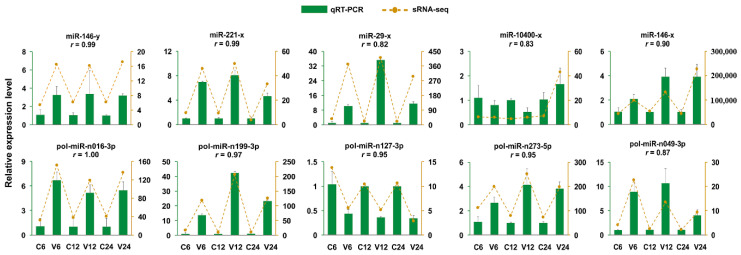
Validation of differentially expressed miRNAs (DEmiRs) by qRT-PCR. The expression profiles of 10 DEmiRs were evaluated by qRT-PCR and compared with that obtained by sRNA-seq. Results are shown as means ± standard deviation (*n* = 3). For each DEmiR, the correlation coefficient (*r*) between the results of qRT-PCR and sRNA-seq is indicated.

After *V. anguillarum* infection, 206 miRNAs showed differential expressions (named DEmiRs) at three time points, among which 104 are novel miRNAs. As shown in Figure 2a, 99 (71 up- and 28 down-regulated), 63 (37 up- and 26 down-regulated), and 95 (52 up- and 43 down-regulated) DEmiRs were identified at 6 hpi, 12 hpi, and 24 hpi, respectively. The top 15 up-/down-regulated DEmiRs at each time point are also shown in Figure 2a. The expressions of all DEmiRs are shown in a heat map in Figure 2b. Among the 206 DEmiRs, only 12 displayed differential expressions at all three time points (Figure 2c). To verify the DEmiRs obtained by sRNA-seq, the expressions of 10 DEmiRs, including 5 known miRNAs and 5 novel miRNAs, were tested by qRT-PCR. As shown in Figure 3, the results of qRT-PCR were consistent with that of sRNA-seq at 6 hpi, 12 hpi, and 24 hpi, with correlation coefficients ranging from 0.82 to 1.00 (Figure 3).

### 2.3. Identification of the Target Genes of the DEmiRs

**Figure 4:**
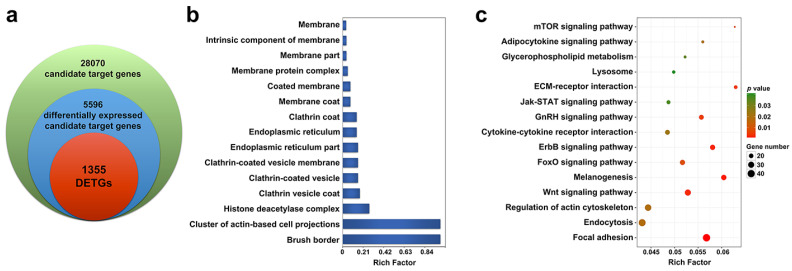
Identification and functional enrichment of the differentially expressed target genes of DEmiRs (DETGs). (**a**) Venn diagram showing the process of DETG identification. Candidate target genes were predicted based on bioinformatics analysis, differentially expressed candidate target genes were identified based on their expression changes in response to *Vibrio anguillarum* infection, and DETGs were identified based on the integrative analysis of miRNA-mRNA expressions. (**b**) Gene Ontology (GO) enrichment of DETGs. (**c**) Kyoto Encyclopedia of Genes and Genomes (KEGG) enrichment of DETGs.

A total of 28,070 putative target genes were predicted for the 206 DEmiRs based on the overlapping results of three different methods. From these putative target genes, 5596 differentially expressed candidate target genes were further identified based on their expression patterns in response to *V. anguillarum* infection. The 5596 genes were further submitted to integrated analysis by which the expressions of these genes were compared with that of their respective DEmiRs. As a result, 1355 target genes were finally identified, whose expressions were not only significantly regulated by *V. anguillarum* but also negatively correlated to a significant extent with their respective DEmiRs. These 1355 genes were named differentially expressed target genes of DEmiRs (DETGs; Figure 4a).

### 2.4. Functional Enrichment of the DETGs

To examine the biological processes regulated by the DEmiRs, Gene Ontology (GO) and Kyoto Encyclopedia of Genes and Genomes (KEGG) enrichment analyses were applied to the DETGs. The GO enrichment revealed that DETGs were mainly associated with clathrin-related components, including clathrin-coated vesicle, clathrin-coated vesicle membrane, clathrin vesicle coat, and clathrin coat, as well as the elements of membrane, including membrane protein complex, membrane coat, intrinsic component of membrane, membrane part, and endoplasmic reticulum (Figure 4b). KEGG analysis showed that among the significantly enriched pathways were those highly related to immunity, including endocytosis, cytokine–cytokine receptor interaction, Jak-STAT signaling, lysosome, and mTOR signaling (Figure 4c). Signal transduction pathways, such as FoxO signaling, Wnt signaling, and ErbB signaling, were also enriched. Other enriched functional processes included the pathways associated with endocrine system (melanogenesis, GnRH signaling, and adipocytokine signaling), metabolism (glycerophospholipid metabolism), and cellular motility (focal adhesion and regulation of actin cytoskeleton).

### 2.5. Immune-Related DEmiR-DETG Network Analysis

**Figure 5:**
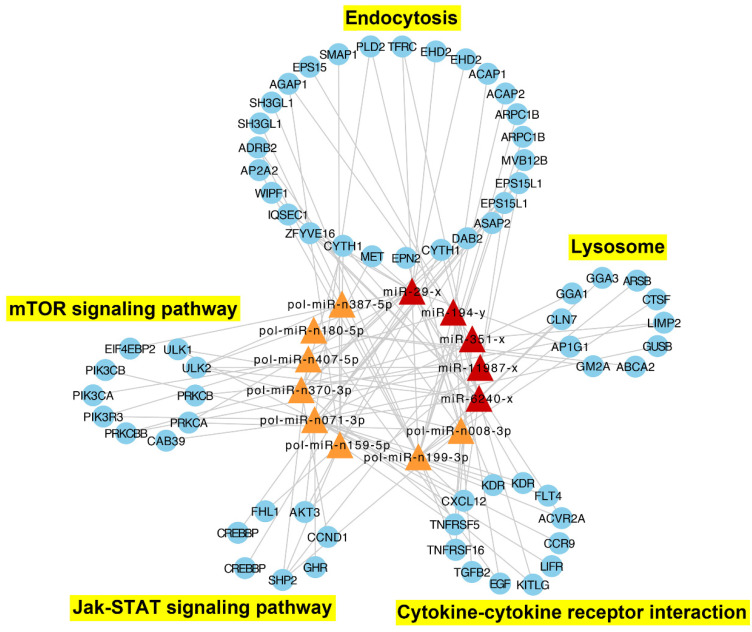
The immune-related network formed by interactive DEmiRs (differentially expressed miRNAs) and DETGs (differentially expressed target genes of the DEmiRs). The blue round nodes indicate immune-related targeted DETGs; the triangle nodes indicate DEmiRs, of which the known miRNAs are in red and the novel miRNAs are in orange. The pathways to which the DETGs are enriched are indicated and marked in yellow.

To gain insights into the functions of the DEmiR-DETG pairs in the immune response to *V. anguillarum* challenge, an immune related DEmiR-DETG network was constructed based on the result of KEGG functional enrichment. The network was composed of 13 key DEmiRs and 66 DETGs, which formed 106 interacting pairs spanning five immune-related pathways (Figure 5). Nine of the 13 key DEmiRs, i.e., pol-miR-n407-5p, pol-miR-n180-5p, pol-miR-n008-3p, miR-194-y, miR-351-x, pol-miR-n370-3p, miR-11987-x, miR-6240-x, and pol-miR-n387-5p, were among the top 15 up-/down-regulated DEmiRs (Figure 2a). Eight of the 13 key DEmiRs are novel miRNAs identified in this study. Of these novel miRNAs, pol-miR-n199-3p was upregulated at all three time points and has multiple DETGs including the TNF-receptor super family member (TNFRSF) 5 and TNFRSF16, cathepsin F (CTSF), ceroid-lipofuscinosis neuronal protein 7 (CLN7), and transferrin receptor (TFRC); pol-miR-n407-5p was the 5th most upregulated miRNA at 6 hpi, the most upregulated miRNA at 12 hpi, and the 3rd most downregulated miRNA at 24 hpi, and targeted protein tyrosine phosphatase non-receptor type 11 (PTPN11, also named as SHP2); pol-miR-n008-3p was the most downregulated miRNA at both 6 hpi and 24 hpi, and targeted phospholipase D2 (PLD2); pol-miR-n370-3p was the 2nd most downregulated miRNA at 12 hpi and targeted hepatocyte growth factor receptor (MET).

**Figure 6:**
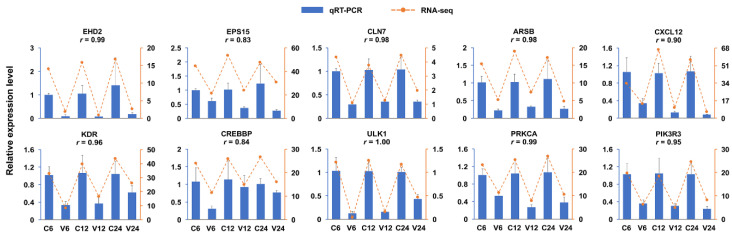
Validation of differentially expressed target genes of the DEmiRs (DETGs) in the immune-related network. The expressions of 10 DETGs enriched in 5 immune-related pathways were tested by qRT-PCR and compared with that obtained by RNA-seq. The results are shown as means ± standard deviation (*n* = 3). For each DETG, the correlation coefficient (*r*) between the results of qRT-PCR and RNA-seq is indicated.

Of the five known miRNAs, miR-29-x was upregulated at all three time points and targeted two EH domain-containing protein 2 (EHD2) genes; miR-194-y was the 2nd most upregulated miRNA at 12 hpi and targeted epidermal growth factor receptor substrate 15 (EPS15); miR-351-x was the 4th most downregulated miRNA at 12 hpi and targeted lysosomal integral membrane protein type 2 (LIMP2); miR-11987-x was the 4th most upregulated miRNA at 24 hpi and targeted unc-51 like autophagy activating kinase 2 (ULK2) and CLN7; miR-6240-x was the 7th most upregulated miRNA at 24 hpi and targeted ULK1/2 and C-X-C motif chemokine ligand 12 (CXCL12; Figure 5). The expressions of 10 DETGs in the network, including EHD2 and EPS15 enriched in endocytosis, CLN7 and ARSB enriched in lysosome, CXCL12 and KDR enriched in cytokine–cytokine receptor interaction, CREBBP enriched in Jak-STAT signaling, and ULK1, PRKCA, and PIK3R3 enriched in mTOR signaling pathway, were validated by qRT-PCR, which showed that the results of qRT-PCR were in agreement with that of RNA-seq [23], with correlation coefficients ranging from 0.83 to 1.00 (Figure 6).

### 2.6. The Networks Formed by DEmiRs and V. anguillarum-Induced Hub Genes

**Figure 7:**
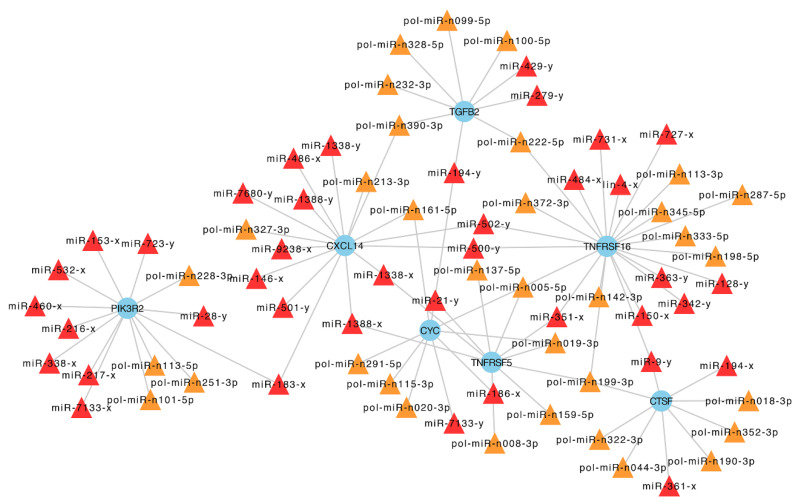
The regulatory networks formed by DEmiRs (differentially expressed miRNAs) and the hub genes identified in an mRNA transcriptome analysis of flounder infected with *Vibrio anguillarum*. The blue round nodes indicate hub genes; the triangle nodes indicate the DEmiRs that target the hub genes, with the known miRNAs in red and the novel miRNAs in orange.

In a previous study, 26 hub genes of flounder highly regulated by *V. anguillarum* infection have been reported [23]. Here, we found that seven of the 26 hub genes, i.e., TNFRSF5, TNFRSF16, CTSF, cytochrome c-b (CYC), CXCL14, phosphatidylinositol 3-kinase regulatory subunit beta (PIK3R2), and transforming growth factor beta-2 (TGFB2), were targeted by 73 DEmiRs. The hub genes and DEmiRs formed extensive networks consisting of 89 interactive pairs that displayed inter-connective relationships with each other, especially among those centering around the hub genes of TNFRSF5, TNFRSF16, CTSF, CYC, CXCL14, and TGFB2 (Figure 7).

## 3. Discussion

In the present study, we examined the micro-transcriptome of flounder in the spleen, one of the major immune organs of teleost fish [24], at different time points during *V. anguillarum* infection. We found that, of the 206 DEmiRs identified, more than 94% (194) exhibited time-specific expression patterns, and among the top 15 up-/down-regulated DEmiRs at each time point, only two miRNAs occurred at more than one time point. These results indicate a highly temporal response of the miRNAs to *V. anguillarum* challenge. At each time point, especially at the early infection stage (6 hpi), more upregulated DEmiRs than downregulated DEmiRs were detected. This observation is in contrast to that found in flounder infected with another bacterial pathogen, *Edwardsiella tarda*, in which most DEmiRs were downregulated upon *E. tarda* stimulation [13]. These results suggest a pathogen specific response of the miRNAs in flounder. Since many genes have been shown to exhibit tissue specific expression, the miRNA profiles identified in this study are likely spleen specific, and may differ at least in some aspects from that in other tissues.

Based on miRNA-mRNA integrative analyses, 1355 DETGs were identified for the 206 DEmiRs. GO and KEGG analyses indicated that the DETGs were significantly enriched in various immune processes. GO is the world’s largest source of information on the functions of genes across multiple species [25]; KEGG is a resource for understanding high-level functions and utilities of biological system from molecular-level information, especially large-scale datasets generated by high-throughput sequencing [26]. Currently, both GO and KEGG databases are mostly supported by the genomes and gene functions of mammals and non-fish animals, which is a disadvantage for fish gene analysis. Nevertheless, GO and KEGG are widely accepted analysis tools and have been used widely in the analysis of mRNA and miRNA transcriptome in fish [9,10,12,13,14]. In our study, 4 of the 15 enriched GO terms were found to be associated with clathrin, a key molecule in the process of clathrin-mediated endocytosis [27]. During bacterial infection, endocytosis can serve as a means to bring the bacteria into the cell, where the bacteria are subsequently killed in the lysosome [28]. The highly enriched clathrin-related components in our study suggest that clathrin-mediated endocytosis likely plays an important role in the cellular ingestion of *V. anguillarum*. Previous reports have shown that cytokines are pivotal modulators of inflammatory responses by activating the Jak-STAT cascade [29,30]. Cross-talks between cytokine and mTOR signaling pathways control inflammatory responses after pathogen infection [31]. Moreover, mTOR also regulates autophagy, a process that contributes to intracellular defense against bacterial invasion [32]. In fish, *V. anguillarum* has been shown to induce miRNAs targeting the genes related to cytokine–cytokine receptor interaction and mTOR in turbot and miiuy croaker [11,33]. In our study, we found that the DETGs were over-represented in the pathways of cytokine–cytokine receptor interaction, Jak-STAT, and mTOR signaling, suggesting inductions of inflammatory response and mTOR-associated autophagy in flounder during *V. anguillarum* infection.

Like all physiological processes, immune response is modulated by gene interaction networks [34]. In our study, immune-related DEmiR-DETG networks were found to be formed by 13 key DEmiRs and their DETGs, some of the DETGs being identified both in this study and in a previous mRNA expression study as the hub genes induced by *V. anguillarum* [23]. It is notable that 8 of the 13 key DEmiRs are novel miRNAs. The abundance of novel miRNAs is a striking feature of our study. We found that novel miRNAs accounted for 39% of the total miRNAs, 50% of the DEmiRs, and 62% of the key DEmiRs in the networks. This observation highlights the importance of novel miRNAs in *V. anguillarum*-induced host response, especially that associated with immunity. Of the eight novel miRNAs in the DEmiR-DETG networks, pol-miR-n199-3p was upregulated at all three time points and targeted five genes, i.e., TFRC associated with endocytosis, CLN7 associated with lysosome, and three immune related hub genes (TNFRSF5, TNFRSF16, and CTSF) identified previously [23]. Of these target genes, TFRC traffics from the cell surface to early recycling endosome, and was reported to be manipulated by a mouse mammary tumor virus for entry into host cells [35]; CLN7 is a membrane protein located in lysosome, and its deficiency leads to lysosomal dysfunction and impaired autophagy in mice [36]. The significantly upregulated expression of pol-miR-n199-3p observed in our study implies a systematic repression of all the target genes, which likely has a significant effect on *V. anguillarum* infection. It remains to be investigated whether repression of these target genes facilitates host clearance of the bacterial pathogen or, as a result of bacterial manipulation of the host immune response, promotes the invasion of the pathogen. Of the other novel miRNAs in the DEmiR-DETG networks, pol-miR-n407-5p was predicted to target SHP2, which modulates Jak-STAT signaling and is required for effective inflammatory response and clearance of *Haemophilus influenza* in mice [37,38]. Two other novel miRNAs, pol-miR-n008-3p and pol-miR-n370-3p, targeted PLD2 and MET, respectively, which have been reported to contribute to the infection and internalization of *Yersinia enterocolitica* and *Listeria monocytogenes*, respectively [39,40]. These results indicate that, through their target genes, the novel miRNAs very likely affect important immune processes involved in pathogen internalization, intracellular trafficking, and clearance.

In addition to the novel miRNAs, the key DEmiRs identified in this study contained five known miRNAs, two of which are miR-29-x and miR-194-y. MiRNAs related to miR-29-x, such as miR-29a, miR-29b, and miR-29b-3p, were shown to be induced by pathogens in half-smooth tongue sole, zebrafish, and Nile tilapia [41,42,43]. Similarly, miR-194 was also regulated by various pathogens in fish [12,41,43,44,45]. In our study, miR-29-x and miR-194-y were stimulated by *V. anguillarum* and predicted to target EHD2 and EPS15, respectively. In mammalian systems, EHD2 is a negative regulator of endocytosis [46], while EPS15 is a component of clathrin-coated pits and coated vesicles, which can be recruited by enteropathogenic *Escherichia coli* to usurp host endocytic machinery [47,48]. These observations suggest a role of miR-29-x and miR-194-y in the modulation of endocytosis during *V. anguillarum* infection in flounder. Of the other key DEmiRs identified in our study, miR-351 is involved in anti-virus (hepatitis C virus) and anti-parasite (schistosomiasis) activities in mammals, and suppresses PTEN expression to promote inflammation [49,50,51]. In fish, no target of miR-351 has been reported. In our study, miR-351-x was downregulated and targeted LIMP2. In rat, LIMP2 is present in the membranes of lysosomes and late endosomes, and contribute to the uptake, killing, and digestion of microbes [52]. In fish, LIMP2 was detected in the immune response to *V. anguillarum* in turbot, but its function is unclear [53]. The downregulated miR-351-x in our study suggests a possibly elevated activity of lysosome that may facilitate *V. anguillarum* degradation.

The other two miRNAs among the 13 key DEmiRs are miR-11987-x and miR-6240-x. To date, the study on these miRNAs is very limited. In our study, miR-11987-x interacted with CLN7, which functions in autophagy [36], while miR-6240-x was found to target ULK1 and interact with ULK2. ULK1 and ULK2 are autophagy induction proteins in mammals and essential for antiviral responses through IFN signaling and for bacterial clearance by autophagy through the mTOR signaling pathway [54,55,56]. In our study, miR-6240-x also targeted CXCL12, which is crucial to neutrophil trafficking [57]. Hence, miR-6240-x was involved in both cytokine and mTOR signaling, the cooperation of which has been shown to be vital for innate immune homeostasis in mice [31]. These observations indicate that flounder miR-11987-x and miR-6240-x, through interaction with CLN7, ULK1/2, and CXCL12 may participate in autophagy and regulation of innate immune homeostasis during *V. anguillarum* infection.

## 4. Materials and Methods

### 4.1. Sample Preparation

Biological samples used in this study have been described previously [23]. Briefly, clinically healthy Japanese flounder (214.7 ± 15.2 g) were purchased from a local fish farm and acclimatized in the laboratory for one week [23]. *V. anguillarum* C312, a pathogenic strain isolated from diseased flounder [58], was cultured in at 28 °C to logarithmic stage, washed with PBS, and resuspended in PBS as reported previously [23]. Fish were randomly divided into two groups named V (*V. anguillarum*-infected group) and C (uninfected control group). The fish in group V were injected intramuscularly with *V. anguillarum* (10^8^ CFU/fish), and the fish in group C were similarly injected with PBS [23]. For each group, three fish were sampled at 6 h post-infection (hpi), 12 hpi, and 24 hpi, and at each time point, spleen was dissected aseptically and used for transcriptome analysis [23]. In total, 18 samples (9 from V group and 9 from C group) were prepared for transcriptome analysis. The experiments involving live fish were approved by the Ethics Committee of Institute of Oceanology, Chinese Academy of Sciences (21 September 2018) (permit No. MB1809).

### 4.2. Small RNA Library Construction and Sequencing

Total RNAs were extracted from the 18 fish samples using Trizol RNA extraction reagent (Invitrogen, Carlsbad, CA, USA) following the manufacturer’s protocol. The quality of the isolated RNAs was evaluated using NanoDrop Spectrometer ND-2000 (Thermo Fisher Scientific, Waltham, MA, USA) and Agilent 2100 bioanalyzer (Agilent Technologies, Palo Alto, CA, USA), and the RNA with RNA integrity number (RIN) ≥8.8 was used for generating sequencing libraries. The sequencing libraries were constructed following the Illumina’s standard protocol. Briefly, RNA molecules with 18–30 nucleotides (nt) in length were enriched through polyacrylamide gel electrophoresis (PAGE). The 3′ and 5′ adapters were added to the RNAs, which were then used for reverse transcription via RT-PCR. PCR products with a size range of 140–160 base pairs (bp) were enriched to construct the cDNA libraries, which were then sequenced with the SE50 strategy on an Illumina HiSeq2500 (Illumina, San Diego, CA, USA) platform.

### 4.3. Data Processing and miRNA Identification

Raw reads obtained from the 18 libraries were subjected to filtering procedures using fastp (v0.12.4) [59] to remove tags containing unknown nucleotide (“N”), without 3′ adapters or insert small RNA fragments, contaminated with 5′ adapters, containing ploy (A), or shorter than 18 nt. The remaining clean tags were candidate small RNA sequences, the quality of which was evaluated with FastQC (v0.11.8) [60]. The tags passed the quality control were aligned to the small RNAs (sRNAs) in GeneBank database (release 209.0) and Rfam database [61] via Blastall (v2.2.25) with identity >97% [62], and to the reference genome of flounder (GenBank project accession PRJNA369269) via Bowtie (v1.1.2) [63]. These alignments further removed the sRNAs including rRNA, scRNA, snoRNAs, snRNA, tRNA, and small fragments mapped to repeat sequences and degraded from mRNAs. The remaining tags were searched against miRBase (release 21.0) to identify known miRNAs. Meanwhile, novel miRNAs were predicted according to their genome positions and hairpin structures using the software MIREAP (v0.2) [64]. The raw data of sRNA-seq containing 18 fastq files were deposited at the Sequence Read Archive (SRA) in NCBI with the accession number of SRP241633.

### 4.4. Differential Expression Analysis

The expression levels of the identified miRNAs were calculated and normalized to TPM (tags per million). The matrix of correlations for miRNA expressions between samples was analyzed using R (v3.5.2). Differential expression analysis of the miRNAs was performed using the package edgeR (v3.12.1) with default parameters [65], and miRNAs with *p* < 0.05 and log_2_|FC| > 1 were considered as differential expressed miRNAs (DEmiRs).

### 4.5. Experimental Validation of DEmiRs

To confirm the expression results obtained by sRNA-seq, qRT-PCR was carried out to examine the expressions of 10 randomly selected known and novel miRNAs with differential expressions as reported previously [13]. Briefly, RNA was isolated from the spleen of the fish in group C and group V at 6 hpi, 12 hpi, and 24 hpi (three fish/time point). cDNA synthesis was performed using miRNA first-strand cDNA synthesis kit (Vazyme, Nanjing, China) with stem-loop reverse transcription primers according to the manufacturer’s protocols. To confirm the expression profiles of DETGs, qRT-PCR was conducted for 10 DETGs in the immune-related network as reported previously [23]. qRT-PCR was performed with SYBR Premix Ex Taq II (TaKaRa, Dalian, China) using QuantStudio 3 Real-Time PCR Systems (Thermo Fisher Scientific, CA, USA) according to the manufacturer’s protocol. The expression of each miRNA was normalized to that of 5S rRNA with 2^−ΔΔCt^ comparative Ct method as reported previously [13]. The expression level of each DETG was normalized to that of the reference gene TUBA as reported previously [23]. The PCR primers are list in Table 2. Correlations of qRT-PCR and sRNA-seq/RNA-seq results were analyzed using cor.test in R (v3.5.2) [66].

### 4.6. Identification and Functional Enrichment Analysis of the Target Genes of DEmiRs

The candidate target genes for DEmiRs were predicted using three software, i.e., RNAhybrid (v2.1.2) + svm_light (v6.01), Miranda (v3.3a), and TargetScan (v7.0), with default parameters. The overlapped targets predicted by the three algorithms were considered as the candidate target genes of DEmiRs. To be stringent, the candidate target genes were subjected to integrated analysis of miRNA-mRNA expressions using the miRNA expression data in this study and the mRNA expression data reported previously for the same samples [23]. Firstly, R (v3.5.2) was employed to screen the candidate target genes to identify differentially expressed genes induced by *V. anguillarum* infection. Secondly, correlation analysis was performed with cor.test in R (v3.5.2) to identify the differentially expressed genes that exhibited negative correlations with their corresponding miRNAs. As a result, only the genes that were not only differentially expressed but also negatively correlated with DEmiRs in expression were considered as the target genes of DEmiRs. These genes were named differentially expressed target genes of DEmiRs (DETGs).

Gene Ontology (GO) and Kyoto Encyclopedia of Genes and Genomes (KEGG) functional enrichment analyses were carried out based on the GO database [25] and KEGG database [26], respectively. Hypergeometric test was performed to evaluate the *p* value, and *p* < 0.05 was set as the threshold for significantly enriched GO terms and KEGG pathways.

### 4.7. Interaction Network Construction

The DEmiRs that targeted the DETGs from the immune-related KEGG pathways and exhibited expression fold change >8 at more than one time point were identified as key DEmiRs. The key DEmiRs and their target DETGs were used to construct the immune-related networks with Cytoscape (v3.7.1) [67]. The networks formed by the 26 hub genes of flounder induced by *V. anguillarum* infection [23] and their target DEmiRs were constructed similarly.

## 5. Conclusions

This study provides the first high-quality global micro-transcriptome of flounder in response to *V. anguillarum* infection. We identified 206 DEmiRs, 50% of which are novel miRNAs identified for the first time. The DEmiRs exhibited highly time-specific expression patterns and were predicted to target 1355 DETGs. Thirteen key DEmiRs and their corresponding DETGs formed extensive regulatory networks associated with five immune-related pathways. These results reveal a deep involvement of miRNAs in the immune response of flounder induced by *V. anguillarum*, and add new insights into the roles of miRNAs in fish.

## Figures and Tables

**Table 1:** Summary of the micro-transcriptome data of flounder infected with and without (control) *Vibrio anguillarum* for different hours.

Sample	Raw Reads	Clean Tags	Clean Tag Ratio (%)	Known miRNAs	Novel miRNAs
C6-1	16,527,156	15,539,755	94.03	15,085,700	46,727
C6-2	18,554,480	17,416,884	93.87	16,756,252	52,469
C6-3	17,117,852	15,872,573	92.73	14,743,097	73,357
V6-1	13,573,207	12,587,907	92.74	12,020,280	42,670
V6-2	13,670,487	12,833,698	93.88	12,402,383	38,667
V6-3	14,740,487	13,869,084	94.09	13,340,657	43,309
C12-1	17,537,783	16,514,129	94.16	15,871,998	35,761
C12-2	18,318,853	17,325,699	94.58	16,771,261	39,827
C12-3	16,878,025	15,949,928	94.50	15,557,458	41,047
V12-1	14,261,792	13,445,075	94.27	13,026,452	38,849
V12-2	13,024,065	12,137,610	93.19	11,548,284	38,653
V12-3	13,932,272	13,099,501	94.02	12,708,381	30,701
C24-1	14,483,183	13,775,520	95.11	13,434,606	31,314
C24-2	16,345,997	15,415,782	94.31	14,832,918	36,044
C24-3	15,877,162	15,046,540	94.77	14,680,203	35,409
V24-1	13,696,884	12,723,780	92.90	12,196,940	29,841
V24-2	14,965,045	13,559,891	90.61	12,035,144	23,242
V24-3	13,356,165	11,772,273	88.14	6,840,857	19,745

For convenience, “C6”, “C12”, and “C24” indicate the control groups at 6, 12, and 24 h post-infection (hpi), respectively; “V6”, “V12”, and “V24” indicate the *V. anguillarum*-infected groups at 6, 12, and 24 hpi, respectively. For each group at each time point, three individual fish were used as biological triplicates.

**Table 2:** List of primers used for stem-loop RT-PCR and qRT-PCR in this study.

MicroRNA	Primer	Sequence (5′ to 3′)
MiR-146-y	Stem-1	GTCGTATCCAGTGCAGGGTCCGAGGTATTCGCACTGGATACGACCAAAAG
	M-146y-f	GCGCGTCTATGGGCTTAGTT
	M-146y-r	AGTGCAGGGTCCGAGGTATT
MiR-221-x	Stem-2	GTCGTATCCAGTGCAGGGTCCGAGGTATTCGCACTGGATACGACACAGAA
	M-221x-f	GCGACCTGGCATACAATGTAGAT
	M-221x-r	AGTGCAGGGTCCGAGGTATT
MiR-29-x	Stem-3	GTCGTATCCAGTGCAGGGTCCGAGGTATTCGCACTGGATACGACTCTATG
	M-29x-f	GCGCTGATTTCATCTGGTGA
	M-29x-r	AGTGCAGGGTCCGAGGTATT
MiR-10400-x	Stem-4	GTCGTATCCAGTGCAGGGTCCGAGGTATTCGCACTGGATACGACCGTCCA
	M-10400x-f	GCGGCGGCGGCGACTC
	M-10400x-r	AGTGCAGGGTCCGAGGTATT
MiR-146-x	Stem-5	GTCGTATCCAGTGCAGGGTCCGAGGTATTCGCACTGGATACGACCCATCT
	M-146x-f	CGCGTGAGAACTGAATTCCAT
	M-146x-r	AGTGCAGGGTCCGAGGTATT
Pol-miR-n016-3p	Stem-6	GTCGTATCCAGTGCAGGGTCCGAGGTATTCGCACTGGATACGACCAAAAG
	M-016-f	GCGCGATCTATGGGCTTAGTT
	M-016-r	AGTGCAGGGTCCGAGGTATT
Pol-miR-n199-3p	Stem-7	GTCGTATCCAGTGCAGGGTCCGAGGTATTCGCACTGGATACGACGCCAGC
	M-199-f	CGCGCAACACTGGTTTGTAA
	M-199-r	AGTGCAGGGTCCGAGGTATT
Pol-miR-n127-3p	Stem-8	GTCGTATCCAGTGCAGGGTCCGAGGTATTCGCACTGGATACGACAGTGCA
	M-127-f	CGCGCCTATGCTTGATTACT
	M-127-r	AGTGCAGGGTCCGAGGTATT
Pol-miR-n273-5p	Stem-9	GTCGTATCCAGTGCAGGGTCCGAGGTATTCGCACTGGATACGACTGTTCA
	M-273-f	CGCAGGACTTGACCCACATG
	M-273-r	AGTGCAGGGTCCGAGGTATT
Pol-miR-n049-3p	Stem-10	GTCGTATCCAGTGCAGGGTCCGAGGTATTCGCACTGGATACGACAGCTAA
	M-049-f	GCGCACCTACCATGTTAGCA
	M-049-r	AGTGCAGGGTCCGAGGTATT
5s	Stem-11	CGGTCTCCCATCCAAGTA
	5s-f	CCATACCACCCTGAACAC
	5s-r	CGGTCTCCCATCCAAGTA
EDH2	edh2-f	CCGCAAACTCAACCCTTTCG
	edh2-r	GGAAGTCATAACCTCGGCTC
EPS15	eps15-f	CCAGCTTAGATGCAGATCCGT
	eps15-r	ACTGGTCAGCCCCATTTGAC
CLN7	cln7-f	CATGTTCGCCTGGACAAGGA
	cln7-r	GGACGATCCCCAACCCTTTG
ARSB	arsb-f	GATTGGCTGCCTACCCTTGT
	arsb-r	GAGGCAAACCCGTAGCTGAT
CXCL12	cxcl12-f	TCGTTCTACCCTCAACACGG
	cxcl12-r	GCTCTTCAGTTTGGCAATGACT
KDR	kdr-f	CAGCTCCACTATGACGACCC
	kdr-r	CTGTGTGACCCTCCATGACG
CREBBP	crebbp-f	GCTGTCACGCAGCTGTCTC
	crebbp-r	TCCTCGGTCTCCATCTTGGT
ULK1	ulk1-f	GTGAGGACACCATTCGGGTT
	ulk1-r	GAAGCCGAAGTCCGCAATCT
PRKCA	prkca-f	CTTCAAGCCCAAAGTGTGTGG
	prkca-r	TCTCATTCGGTGCTGCTGAG
PIK3R3	pik3r3-f	ACTGGTGGAATACAACGCCA
	pik3r3-r	TCATCTGGATTTCCTGTGAGG
TUBA	tuba-f	TGACATCACAAACGCCTGCTTC
	tuba-r	GCACCACATCTCCACGGTACAG
